# Soft and Stretchable Sensor Using Biocompatible Electrodes and Liquid for Medical Applications

**DOI:** 10.1089/soro.2015.0011

**Published:** 2015-12-01

**Authors:** Stefania Russo, Tommaso Ranzani, Hongbin Liu, Samia Nefti-Meziani, Kaspar Althoefer, Arianna Menciassi

**Affiliations:** ^1^ Department of Computer Science, School of Computing, Science and Engineering, The University of Salford, Salford, United Kingdom.; ^2^ The BioRobotics Institute, Scuola Superiore Sant'Anna, Pontedera, Italy.; ^3^ Department of Informatics, The Centre for Robotic Research (CoRe), King's College London, London, United Kingdom.

## Abstract

This article introduces a soft and stretchable sensor composed of silicone rubber integrating a conductive liquid-filled channel with a biocompatible sodium chloride (NaCl) solution and novel stretchable gold sputtered electrodes to facilitate the biocompatibility of the sensor. By stretching the sensor, the cross section of the channel deforms, thus leading to a change in electrical resistance. The functionalities of the sensor have been validated experimentally: changes in electrical resistance are measured as a function of the applied strain. The experimentally measured values match theoretical predictions, showing relatively low hysteresis. A preliminary assessment on the proposed sensor prototype shows good results with a maximum tested strain of 64%. The design optimization of the saline solution, the electrodes, and the algebraic approximations derived for integrating the sensors in a flexible manipulator for surgery has been discussed. The contribution of this article is the introduction of the biocompatible and stretchable gold sputtered electrodes integrated with the NaCl-filled channel rubber as a fully biocompatible solution for measuring deformations in soft and stretchable medical instruments.

## Introduction

Recent years have seen a growing interest in the development of continuum-like and soft robots that can bend, extend, and contract at any point along their length. This flexibility provides them with capabilities well beyond those of their rigid-link counterparts,^1^ thus allowing them to perform whole-arm manipulation. Some examples are a soft robotic arm modeled on the octopus^2^ and a multigait soft walker powered by compressed air.^3^A key use of soft robotics can be found in medical applications, where attention has been currently focused on minimizing the invasiveness of existing minimally invasive surgery (MIS) approaches. In surgery, in particular, one recent approach to soft and modular systems is represented by the ongoing EU project STIFF-FLOP (www.stiff-flop.eu). The STIFF-FLOP arm takes inspiration from biological manipulation and it is fabricated from soft structures showing advanced manipulation capabilities for surgical applications, with multiple degrees of freedom, bending, and ability of elongating and squeezing.^4^

In contrast, these soft and flexible devices introduce new challenges because sensing and controlling the shape and motion of continuum robots are more complicated due to their structural flexibility^5^; in fact, the contact region can be quite wide and is not limited to the end effector tip as for rigid robots.^6^

With regard to sensing, medical robots are expected to smartly perform in unstructured and changing environments, then to work more autonomously and to be more responsive and safe in case of unexpected contacts; furthermore, the surgeons need an interface that can provide information about the manipulator shape inside the body, because the motion is often counterintuitive to human operators, thus generating confusion.

Several sensing strategies for MIS have been proposed over the past years,^7–10^ Ideally, the entire robotic structure should safely move with contact and bend detection and the embedded sensors should not interfere with the overall motion, remaining functional even when highly deformed.^11^ For these reasons, the use of small, soft, and stretchable sensors becomes necessary, especially inside the human body.

Previous approaches in stretchable sensors include, for example, a capacitive sensor made of gold films embedded in silicone rubber,^12^ carbon black-filled silicone,^13^ metal strain gauge incorporated in a flexible polymer substrate,^14^ and tactile sensors with piezoresistive cantilevers embedded into an elastic material.^15^ These devices, despite their flexibility, are not truly stretchable because they use solutions in which rigid sensing elements are integrated in a supporting elastic substrate, thus following but limiting its overall deformation, and, in this way, the rigid part constituting the sensor usually moves inside the soft matrix, thus risking the material failures and affecting the mechanical properties of the material itself; for these reasons they cannot remain functional at large strains.

Other studies include the use of silicone rubber channels filled with conductive liquids. These solutions use a particular type of conductive liquid material, which is eutectic gallium–indium (eGaIn). Some examples are a pressure^16^ and strain^17^ sensor, a structure with multilayered microchannels for detecting multiaxis strains and pressure,^18^ and a bending sensor.^19^ Despite the advantages of using eGaIn as high surface tension and low viscosity, enabling an easy manufacturing process,^20^ the real drawback is that this liquid is not biocompatible^21^; therefore, the exploitation of different conductive fluids is needed for medical applications.

**FIG. 1.**
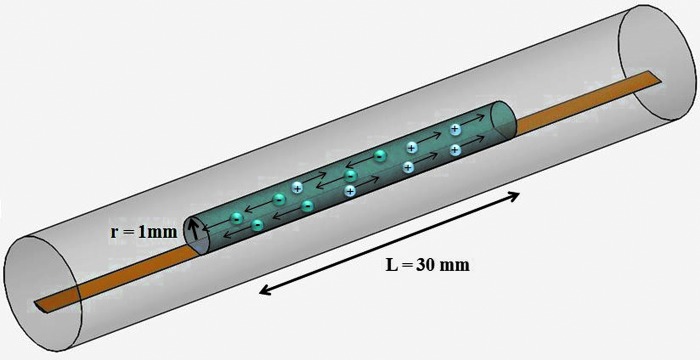
View of the final prototype of the soft sensor: When a voltage difference is applied between the electrodes inside the solution, the Na^+^ and Cl^−^ ions are attracted, respectively, to the negative anode and the positive cathode. As charge carrier, their movement inside the solution generates a current. In gold, the sputtered biocompatible electrodes. Color images available online at www.liebertpub.com/soro

For the aforementioned reasons, we introduce a small, low-cost, soft, and stretchable sensor composed of a silicone rubber (Ecoflex0030; SmoothOn), integrating a conductive liquid channel filled with biocompatible sodium chloride (NaCl) solution (Fig. 1). The advantage of using silicone rubber is its mechanical durability, high flexibility, nontoxicity, chemical stability, and low cost^22^

Furthermore, liquid conductors eliminate the need for rigid electronics and preserve the natural elasticity of the sensor, and the NaCl solution fulfills the need for a biocompatible liquid. However, NaCl liquid presents a low surface tension and requires an AC excitation.

To address the limits of using an ionic liquid as a sensing element, in the next sections we will show our improvements to the ionic solution in terms of its viscosity for ease of fabrication.

In contrast to previous works,^21,23,24^ where nonstretchable nickel–titanium electrodes and nonbiocompatible sensitive liquids were used, in the next sections we will present our solution for achieving a fully biocompatible soft sensor with stretchable and biocompatible electrodes. The possible future implementation of the sensor inside a surgical manipulator will be discussed.

## Concept and System Design

### Design of the sensor

For our first analysis, we chose a rectangular geometry; the channel has a width *w* = 8.8 mm, height *h* = 2.2 mm, length *L* = 38 mm and is located at a distance of 2 mm above the upper surface of the silicone rubber. Being this first study, a proof of concept, these dimensions were chosen mainly for manufacturing easiness.

When strain is applied in the axial direction of the channel, the length increases and the cross-sectional area of the channel decreases, thus causing an increase in the resistance between the electrodes. For the chosen geometry, the theoretical relationship between the resistance change ΔR and strain can be found as follows^18^:

\begin{align*}\Delta R = R - R_0 = \rho { \frac { L + \Delta L }
{ ( w + \Delta w ) ( h + \Delta h ) } } - \rho \frac { L }  { wh }
, \tag { 1 } \end{align*}

where ΔR is the difference between the resistances of the channel when stretched by ΔL and when not stretched, and *ρ* is the resistivity of the solution.

Poisson's ratio *v* is the ratio of transverse contraction to the longitudinal extension strain in the direction of the applied load. For the overall volume constancy, when the channel with diameter (or width, or thickness) *d* and length *L* is subject to stretch so that its length will change by *ΔL* then its diameter *d* will change by

\begin{align*}\Delta d = - d \left( 1 - \left( 1 + \frac { \Delta
L }  { L } \right) ^ { - v } \right) , \tag { 2 } \end{align*}

as we are considering large deformations.

Since $\varepsilon = \frac { \Delta L }  { L }$ and *v* = 0.5 for an elastomer material, it is possible to simplify the above equation as follows:

\begin{align*} \frac { \Delta R }  { R_0 } = \varepsilon ( 2 +
\varepsilon ). \tag { 3 } \end{align*}

### Fabrication

**FIG. 2.**
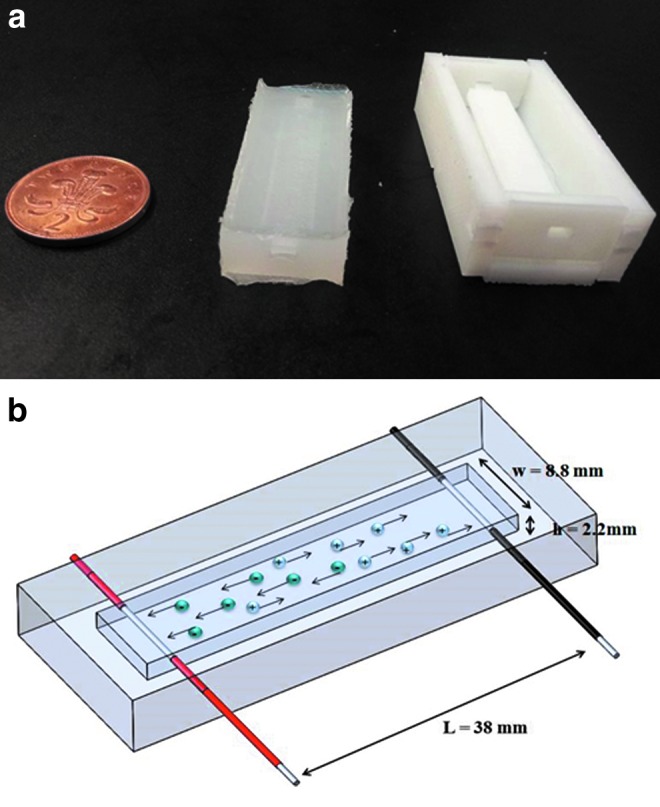
**(a)** On the *right*, view of the first prototype of the mold, and on the *left*, the cured silicone with an embedded channel. **(b)** View of the first prototype of the soft sensor with rectangular geometry. Color images available online at www.liebertpub.com/soro

The sensors are produced by casting silicone rubber (Ecoflex 0030; SmoothOn) into three-dimensional (3D) printed molds (Fig. 2).

The silicone is mixed 1A:1B by weight causing the formation of entrapped air, so vacuum degassing has been used to eliminate air bubbles in cured rubber.

After ∼4 h at room temperature, the elastomer is cured with negligible shrinkage and removed from the molds: the rubber is translucent, soft, and stretchable many times without distortion. The central channel is obtained by removing the mold trough small holes at the edge of the silicone. Next, two aluminum wires used as electrodes are inserted into the ends of the channel, which is then closed with uncured silicone rubber; a syringe is used to fill the embedded channel with NaCl solution while another one is used at the same time to capture and remove the air inside the tube.

Then, uncured rubber is casted for sealing all the holes and preventing the formation of air bubbles.

The NaCl solution is prepared by dissolving 1 g of salt in 10 g of water, thus reaching a concentration of 10%; the resistivity was then calculated from a resistivity table, ensuring that all tests were conducted at a constant temperature of 25°C.

## Experimental Setup and Calibration

**FIG. 3.**
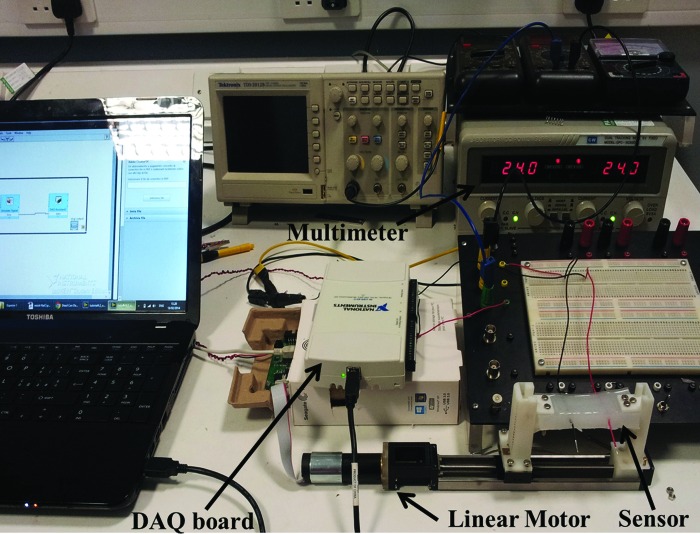
Experimental setup for measuring applied strain and electrical resistance for the sensor prototype. From the *right*, the laptop, the National Instrument data acquisition (NI DAQ) board, the electrical circuit, the multimeter, and the prototype. Color images available online at www.liebertpub.com/soro

The sensor has been experimentally characterized under tensile stress. The experimental setup (Fig. 3) is composed of a linear stage using a HIWIN linear motor, a multimeter, a laptop, and a National Instrument data acquisition (NI DAQ) board.

The HIWIN stage is used for performing accurate stretching tests. Rigid 3D printed clamps are fixed around the edges of the sensor and the motor. Tensile tests were performed by imposing displacements up to 51% from 0 to 35 mm with a step of 1 mm.

A LabVIEW program is used for generating the supply of the sensor, through an analog output of NI DAQ board USB-6211. The generated signal was 500 mV AC voltage with a frequency of 4 kHz; the use of an ionic solution introduces constraints in the design of the associated electrical circuit since using a DC could cause the polarization of the electrodes. To avoid this problem, an AC excitation is used in combination with a low voltage sine wave, to avoid electrolysis of the fluid.^25^

Furthermore, a 4 kHz frequency was chosen because signals at high frequency are less dangerous since they excite cells in a negligible way.^26^

Changes in electrical resistance are then calculated from the measurement of the current generated at the electrodes.

The expected resistance of the filled channel was estimated as R = 129 Ω, considering the resistivity and the prototype channel dimensions, but the measured one at rest was approximately five times larger due to the high resistance introduced by small air bubbles in the channel. Because of this, for a better comparison between tests, the results of each test have been offset by their initial resistance.

The changes in the electrical resistance were then evaluated by applying axial strain. We produced five prototypes, and each one was gradually stretched; data for each sensor were collected from five stretching tests, although the first one was not used for analytical purpose because rubber-like materials exhibit a different behavior in their mechanical properties resulting from the first extension; this is known as the Mullins effect.^27^

**FIG. 4.**
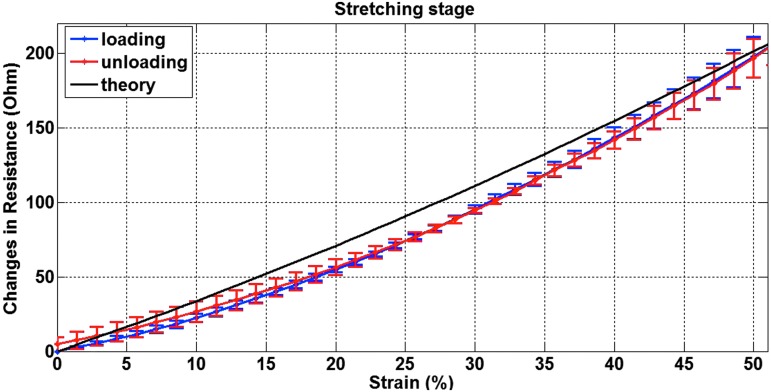
Experimental and theoretical results of changes in resistance are plotted versus strain. Color images available online at www.liebertpub.com/soro

In Figure 4, the average curve of the repeated tests is shown. The repeatability of the sensor was evaluated as the maximum variation between the measurements from five prototypes; it was found to be σ = ±14.25, which is ±6.10% of the resistance at the maximum strain. The higher variability is expressed at the maximum tested elongation probably due to the slip of the sensor from the clamps when reaching that position.

The results from the calibration tests contain an average of the data from multiple loading and unloading stages for each prototype, to characterize and demonstrate the hysteresis, showing good repeatability and little difference between the two curves. The area of the hysteresis loop is 38.35 Ω, while the maximum hysteresis is 4.94 Ω, 21.34% of the resistance at the maximum strain.

## System Improvements

### System integration

As already mentioned, the use of continuum and soft robots needs different models of robot shape and motion and the availability of practical, real-time, sensor-based motion planning algorithms is necessary.

A simplified approach approximates the soft robots with a piecewise constant curvature^28^ composed of finite circular arcs, each with a set of parameters that can be converted in position and orientation information, from the configuration space to the task space of the robot with a purely kinematic mapping.

This can be also applied to multisection robots as the STIFF-FLOP, considering independently each section. The manipulator is composed of multiple identical modules, everyone capable of independent bending in various directions, elongation, and stiffening^29^ Each module is a silicone cylinder with three equally spaced semicylindrical chambers in radial arrangement (the fluidic actuators) and another chamber centrally placed (for the stiffening mechanism).

The inflation of one of the fluidic chamber causes a bending coupled with an expansion in the radial direction; to constrain the lateral deformation, a crimped braided sheath has been introduced around the cylinder, providing a radial constraint and at the same time following the robot movements. When more chambers are inflated together, the robot starts to bend and elongation is finally obtained by activating the three chambers of the same module simultaneously. The typical maximum elongation for every chamber is around 100% (from a rest length of 30 mm up to a length of 60 mm). The central chamber can modulate the module stiffness by granular jamming actuation^29^; it is fabricated-filling coffee powder in a latex membrane, and stiffening is produced using a vacuum pump.

**FIG. 5.**
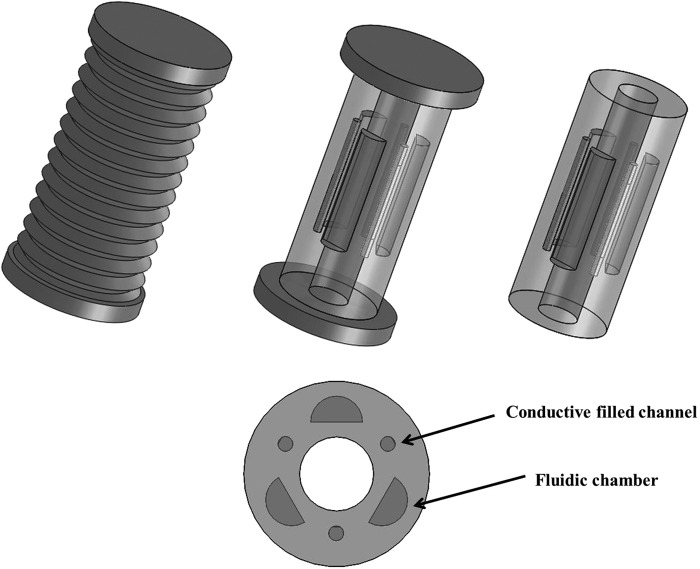
Future integration of the soft sensors inside the STIFF-FLOP robot. At the *bottom*, a section of the silicone module with the three sensors.

For the aforementioned reasons, we propose to use three length sensors inside every silicone module of the STIFF-FLOP robot, equally spaced from the three pneumatic chambers, as shown in Figure 5. Measuring the length of the three chambers (the joint variables *q* = [l1, l2, l3], indicated by the length of three sensors), it is possible to calculate the arc parameters that are a triplet of curvature *k*, the angle of the plane containing the arc *Ø*, and the arc length *l*.

As already mentioned, these sensors have requirements as a result of their application; in fact, they should be soft and stretchable as their host structure to not interfere with the module elongation and bending; sense strain and not pressure inside the chamber when the robot module is bent; furthermore, they should be nontoxic, biocompatible, and small and functional when stretched.

Using all of these information and constraints, we decided to modify the dimensions and the shape of the sensor to improve its response, according to the following analysis.

Park *et al.*^16^ used the linear elastic fracture mechanics and derived a relationship for a channel embedded near the surface of the elastomer, used to evaluate the change in electrical resistance in the pressure mode. Considering *v* = 0.5, the Young's modulus of the used silicone rubber that is 0.6 MPa, and considering to apply a force of about 2 N, with a fingerprint area of 1 cm^2^, so with a total pressure of ∼20 kPa and substituting in the Park *et al.* equation, we obtain

\begin{align*} \frac { \Delta R }  { R_0 } = \frac { 1 }  { ((20
\frac { h }  { \chi w } ) - 1) } \tag { 4 } \end{align*}

where *χ* is a correction depending on the depth *z* of the channel inside the silicone rubber, and *h/w* is the aspect ratio (AR) of the channel.

**FIG. 6.**
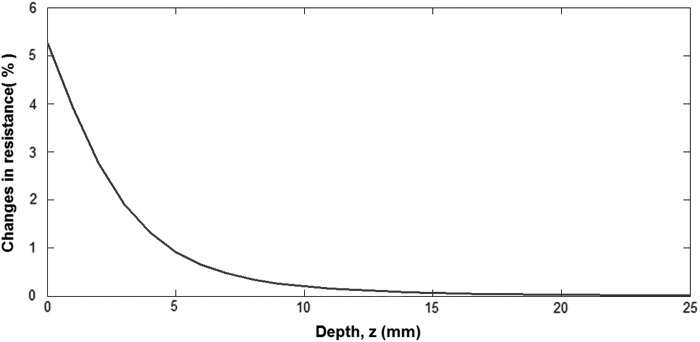
Percentage of changes in sensor electrical resistance function of channel depth when aspect ratio = 1, when considering to apply a total pressure of ∼20 kPa.

As a consequence, it is clear from Equation (4) that if we take into account AR = 1 and a channel deeper in the silicone, the sensitivity to pressure will be smaller; this is shown in Figure 6.

In the stretching mode, if we change the channel geometry to a circular cross section where AR = 1, and considering large deformations, when the channel is subject to stretch ɛ, its radius *r* will change by

\begin{align*}\Delta r = r_f - r_0 = - r_0 ( 1 - ( 1 +
\varepsilon ) ^{ - v} ) \tag{5}\end{align*}

where *r_0_* is the initial radius and *r_f_* is the radius at the maximum elongation.

Considering *v* = 0.5 and the maximum chamber elongation of the STIFF-FLOP robot $\varepsilon = 100\%$, we find

\begin{align*}r_f = 0.707r_0\end{align*}

And the changes in resistance are a function of the initial channel radius:

\begin{align*}\Delta R = R_f - R_0 = \frac { \rho L_f }  { \pi {
r_f } ^2 } - \frac { \rho L_0 }  { \pi { r_0 } ^2 } \ \alpha \
\frac { 1 }  { { r_0 } ^2 } \tag { 6 } \end{align*}

**FIG. 7.**
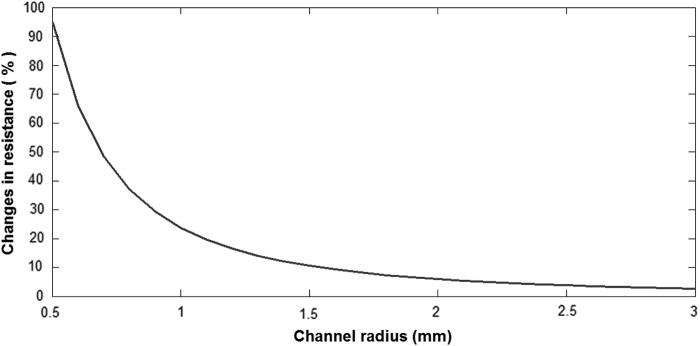
In stretching mode, changes in sensor electrical resistance function of the initial channel radius for a maximum strain of 100%. Using an initial channel radius of 1 mm will maximize the changes in resistance in the stretching mode and will allow the sensor to be functional at large strains as, at the maximum elongation, its radius is reduced by 30%.

For these reasons, we choose as channel dimensions an initial length equal to the STIFF-FLOP fluidic chamber L_0_ = 30 mm, a cylindrical geometry with an AR = 1, and a section radius of 1 mm to maximize the changes in resistance in stretching mode, as shown in Figure 7. These channel dimensions were also chosen for still having a good radius size at 100% elongation, as it is reduced by 30% at the maximum elongation, from Equation (5).

### Air bubble management

Possible reasons for the air bubble formation inside the liquid filled should be searched in the holes created by the electrodes and the high gas permeability of the encapsulating polymer that allows the water to evaporate from the NaCl solution.

For improving the conductive solution, we mixed 65% glycerol with 35% NaCl solution^17^; this dilution was chosen to prevent any undissolved NaCl crystals to be formed and to obtain a more viscous solution for ease of fabrication. As other studies have already demonstrated,^30^ the glycerol is biocompatible and moderate doses do not produce noxious effects as it is widely used as an optical skin clearing agent. In our case, it prevents the formation of bubbles inside the channel.

As expected, the new prototypes did not show air bubbles inside the liquid-filled channel.

### Electrode corrosion

**FIG. 8.**
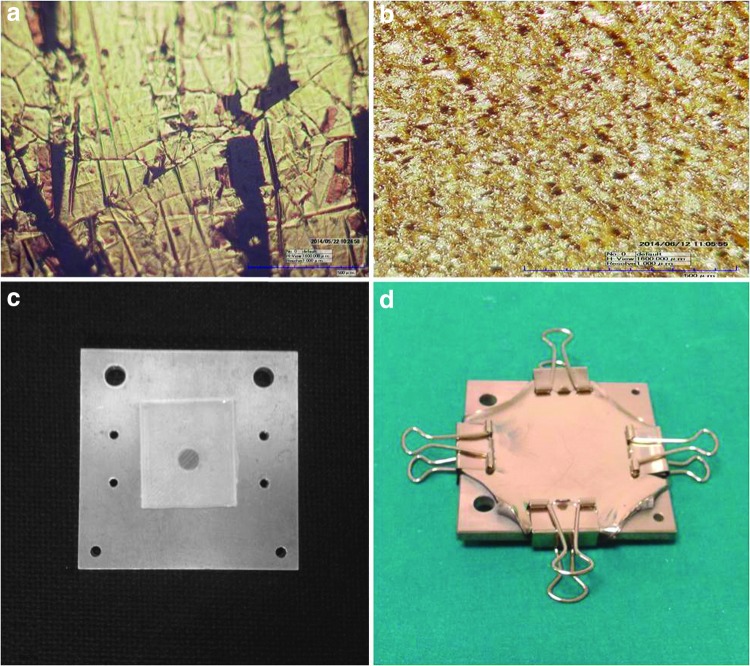
**(a)** Microscope image of the sputtered electrode without prestretching. **(b)** Microscope view of the sputtered wrinkled electrode. **(c)** Silicone rubber before prestretching. **(d)** Silicone rubber after prestretching and sputtering of the metal. Color images available online at www.liebertpub.com/soro

Another challenge was the dimension of the electrodes and their material, which was subjected to corrosion. We decided to produce electrodes through the sputtering deposition of a metal film of 100 nm thickness on the silicone substrate. The sputtered metal was chosen to be gold because it presents a very slow corrosion, and it is biocompatible. Sputtering the metal directly on the substrate without prestretching would create a thin and rigid film, which would be destroyed during the first stretching of the sensor, as shown in Figure 8a, thus not conducting the signal.

For this reason, we decided to perform the sputtering on the silicone with a 200% prestretching to obtain a wrinkled structure when the rubber was released, which could follow the stretching of the sensor^31^ (Fig. 8).

The deposition of the thin biocompatible Au films has been performed by a DCC 150 DC/AC magnetron sputtering system that operates at a constant pressure of 1 Pa. Pure argon with a purity of 99.999% was used as sputtering gas, and the thickness of the gold film was regulated by controlling the deposition time.

The sputtered gold demonstrated conduction during the strain of the silicone sensor, tested with up to 64% deformation.

## System Assessment

**FIG. 9.**
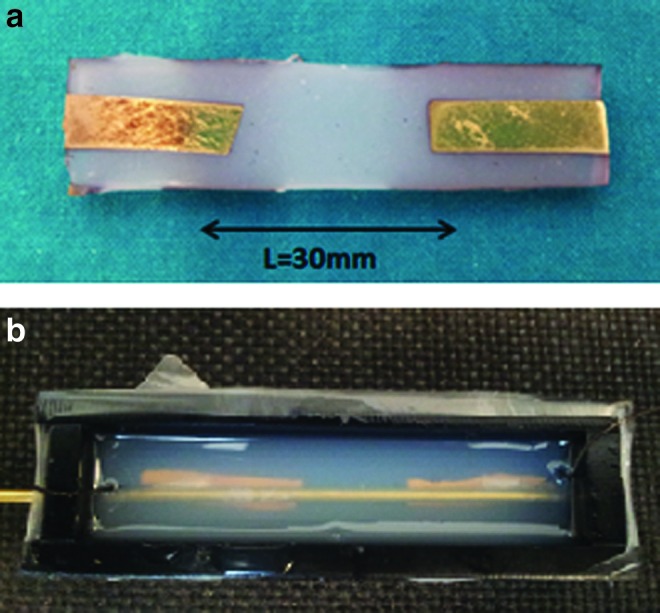
**(a)** On the *top*, the section of the silicone sensor, presenting the sputtered electrodes. **(b)** Silicone casting with the central cylindrical mold. Color images available online at www.liebertpub.com/soro

The new version of the sensor is fabricated in different phases, first creating a thin film by pouring the liquid silicone between two plates: this procedure guarantees a uniform thickness; second, two pieces of gold sputtered silicone are placed on top of the film (as shown in Fig. 9a) and a central cylindrical mold of 1 mm radius is inserted, ensuring the cylinder covers the gold electrodes during the second silicone casting (Fig. 9b). After curing, the central cylindrical mold is removed and the channel closed with uncured silicone rubber; a syringe is then used to fill the embedded channel with the conductive solution while another one is used at the same time to capture and remove the air. Finally, uncured rubber is casted for sealing all the holes, as described for the first prototype fabrication.

To demonstrate the concept and the functionality of the improvements, the cylindrical sensor has been then validated through the Instron testing machine, measuring the voltage output between the gold electrode function of the strain. A maximum of 64% of strain has been reached in this analysis. Above this value, the slipping of the silicone sensor between the machine clamps was creating a nonuniform transfer of the strain. At that maximum extension, the wrinkled structure of the electrodes was still showing conduction.

**FIG. 10.**
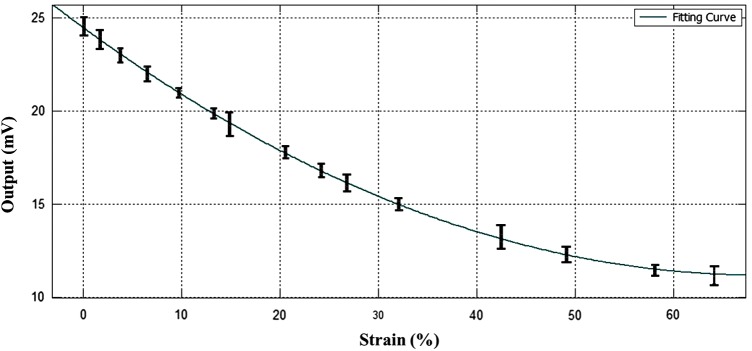
Measured output between the gold electrodes of the cylindrical sensor is plotted versus strain. Color images available online at www.liebertpub.com/soro

The results in Figure 10 show the curve that best fit the experimental data point. This is in reasonable agreement with the theoretical predictions from Equation (3), but further tests need to be carried out to obtain a clear model of the sensor behavior. This suggests that this approach and the principles presented here offer an important contribution to the field of soft sensing in biocompatible devices.

## Conclusions

We presented the proof of concept of a novel soft and stretchable sensor composed of silicone rubber integrated with a biocompatible NaCl solution-filled channel and gold stretchable electrodes.

In contrast to existing soft sensors, the sensor presented here is composed entirely of soft and stretchable materials, with a biocompatible conductive fluid and biocompatible gold sputtered electrodes that make it suitable for medical applications, especially when large deformations are required during the operation of the robot.

Strain of the sensor ɛ is calculated by measuring the change in electric resistance ΔR.

The results showed herein demonstrate a good theoretical matching in strain sensing, and the signal was repeatable, with hysteresis of 21.34%.

This typology of sensing represents just one of the inherent capabilities of this liquid-embedded sensor as sensitivity of the channel is tuneable changing the geometry, as previous works have demonstrated.^32^

We addressed a possible implementation in the STIFF-FLOP surgical manipulator, using the piecewise constant curvature approach. These types of soft robots have the advantage over rigid robots in that they generate little resistance to compressive forces, therefore conforming to unpredictable environments, implying a mechanical self-stabilization and lighter bodies. In contrast, they introduce new challenges, from presenting a sufficiently compliant and controlled interface to being equipped with a sensing strategy that does not interfere with their mechanical properties.

We believe that this work offers an inexpensive and soft solution as a sensing element, which is a key feature for enabling safe and low-cost medical applications. The future developments of this novel technology could potentially contribute to make a fundamental stride in the field of soft and flexible devices in medical applications.

Future study will focus on the improvement and possible customization of the fabrication technique, addressing the integration of this sensor into the ongoing EU project STIFF-FLOP manipulator.
